# Supplementary material for: Engineering twin structures and substitutional dopants in ZnSe0.7Te0.3 anode material for enhanced sodium storage performance
Source: Nat Commun. 2025 May 12;16:4406. doi: 10.1038/s41467-025-59707-0 (PMC12069564; doi:10.1038/s41467-025-59707-0)
Supplement: Supplementary file 1 — Supplementary Information [file 41467_2025_59707_MOESM1_ESM.pdf]

Supplementary Information

Engineering twin structures and substitutional dopants in $ZnSe_{0.7}Te_{0.3}$ anode material for enhanced sodium storage performance

Jingui Zong[1], Fan Liu[1], Yazhan Liang[1], Mingzhe Zhang[1,2], Kepeng Song[1]*, Jinkui Feng[2], Baojuan Xi[1]* & Shenglin Xiong[1]*

[1] School of Chemistry and Chemical Engineering, Shandong University, Jinan 250100, China

[2] School of Materials Science and Engineering, Shandong University, Jinan 250061, China

*email: kpsong@sdu.edu.cn; baojuanxi@sdu.edu.cn; chexsl@sdu.edu.cn

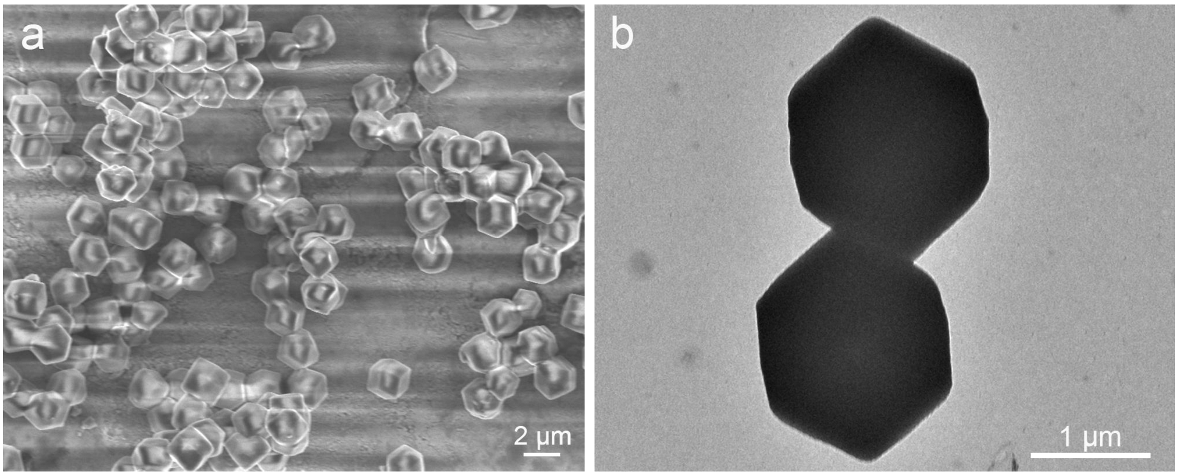

Supplementary Fig. 1 a SEM and **b** TEM images of ZIF-8.

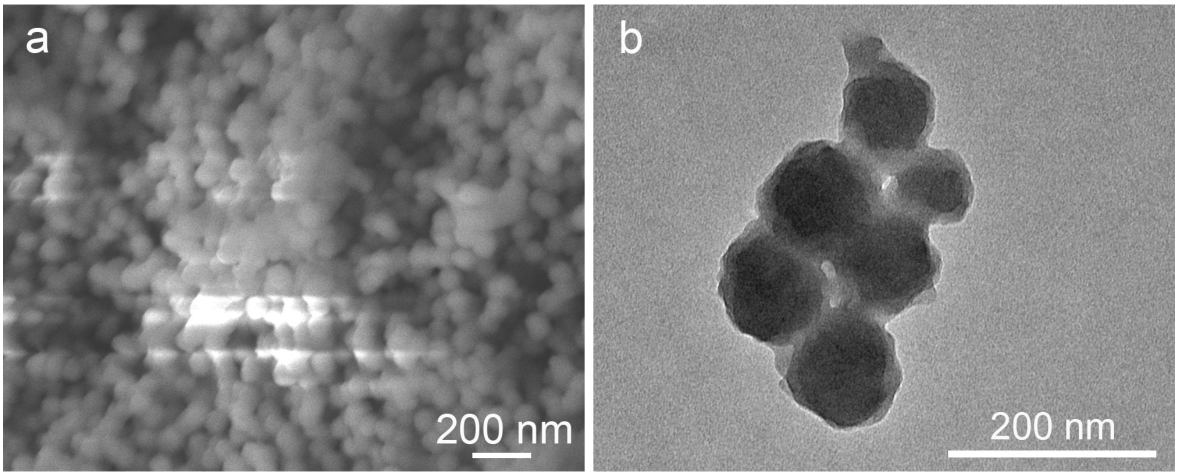

Supplementary Fig. 2 a SEM and **b** TEM images of ZIF-8@MF.

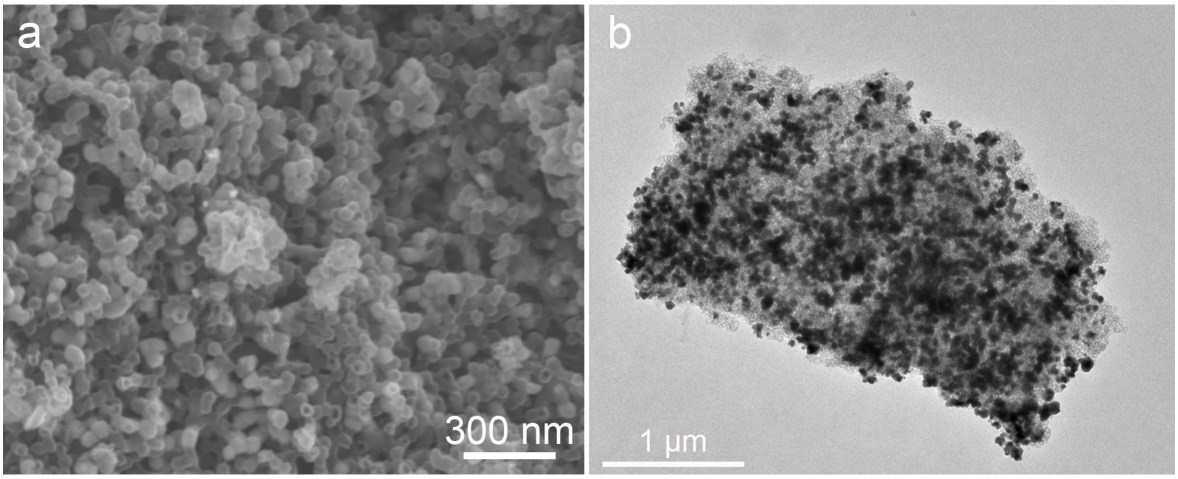

Supplementary Fig. 3 a SEM and **b** TEM images of ZnSe@C.

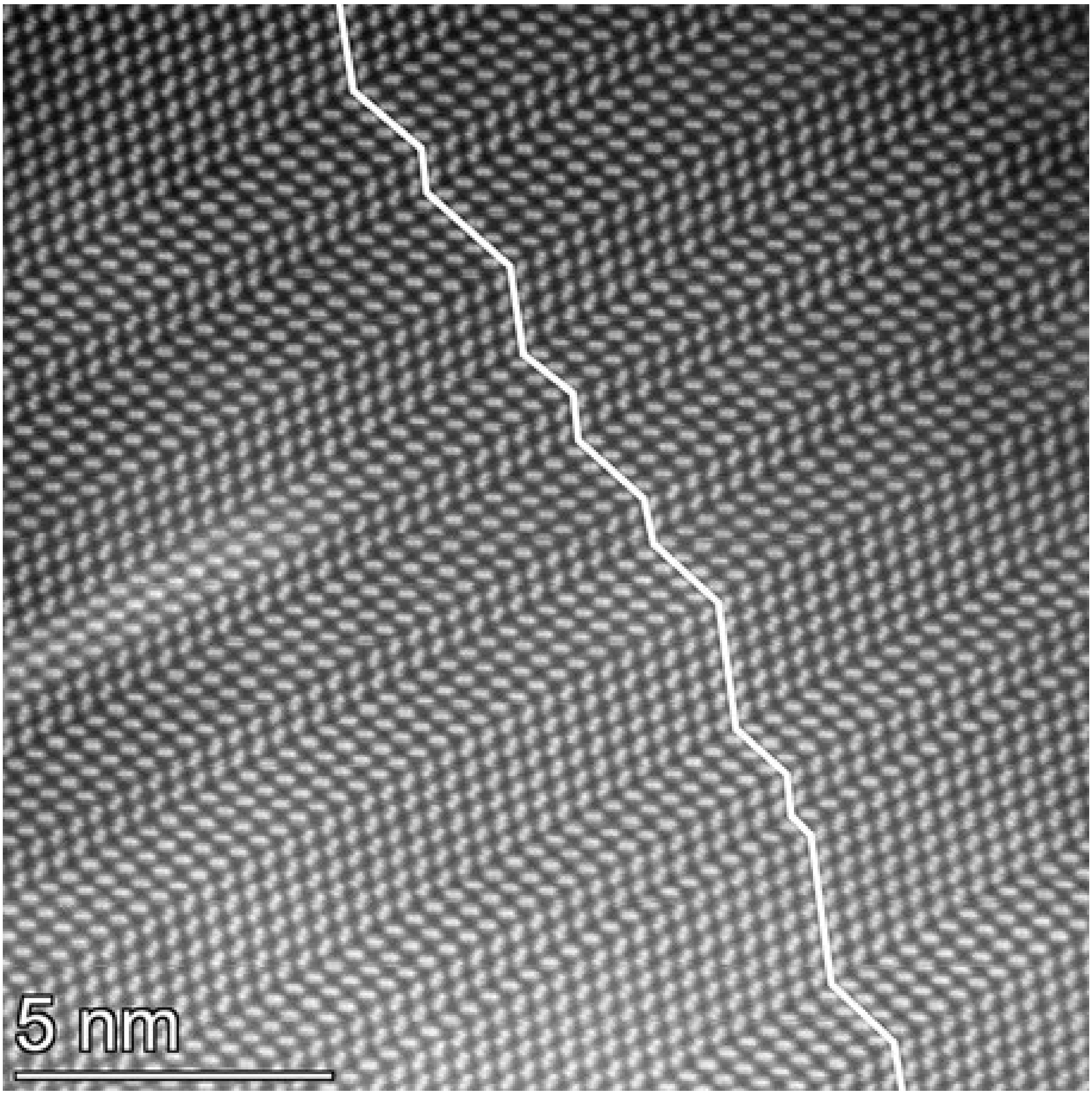

Supplementary Fig. 4 High resolution HAADF-STEM image of $ZnSe_{0.7}Te_{0.3}$@C.

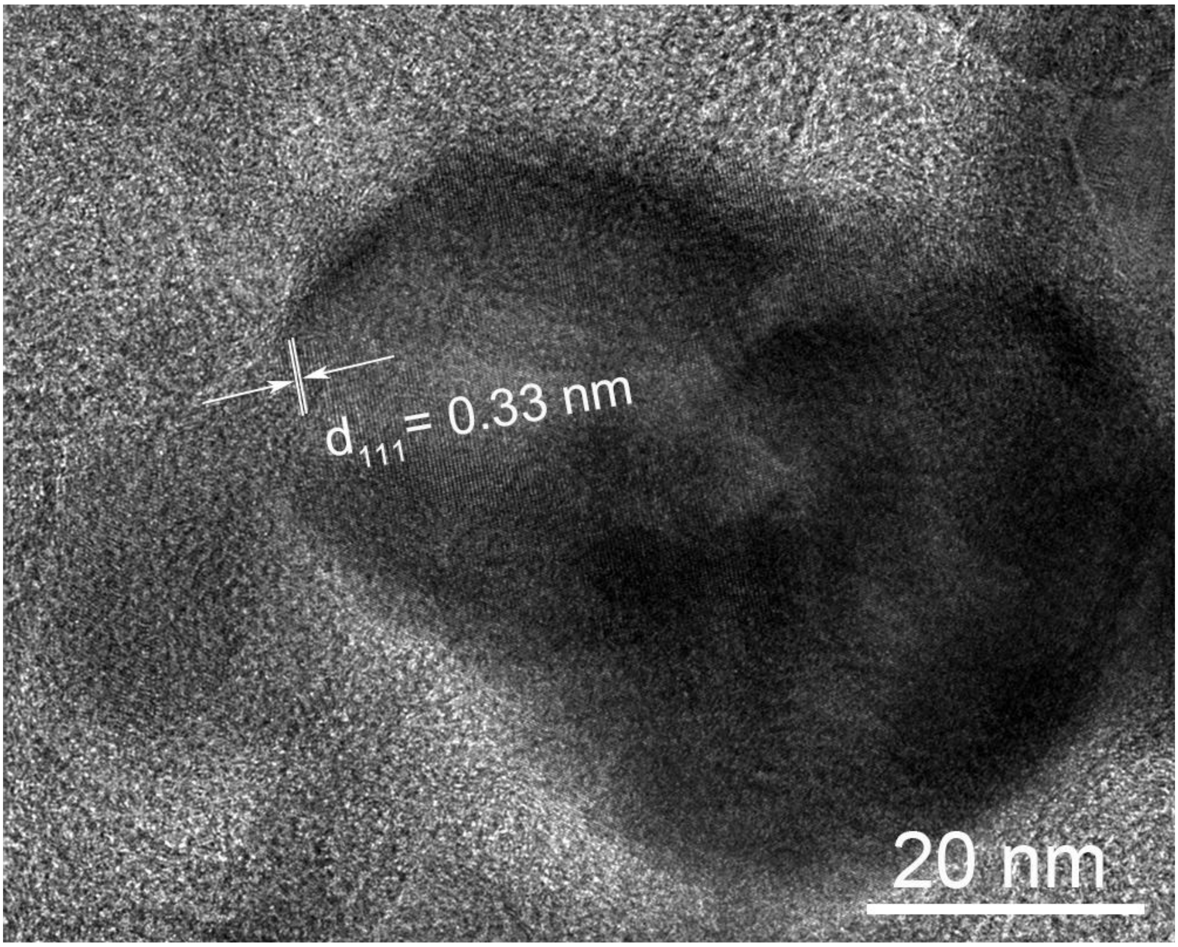

Supplementary Fig. 5 HRTEM image of ZnSe@C.

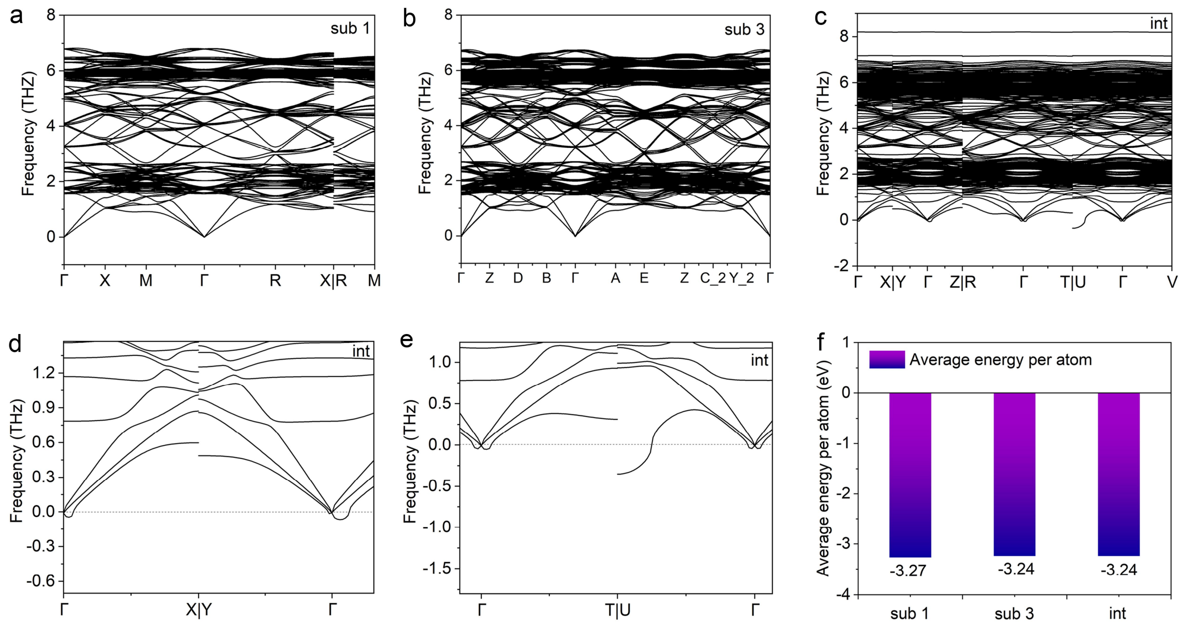

Supplementary Fig. 6 Phonon spectra for the structural models of **a** sub 1, **b** sub 3 and **c** int. **d** and **e** Enlarged view of the phonon spectra for int model. **f** The average energy per atom in the final state of different configuration structures.

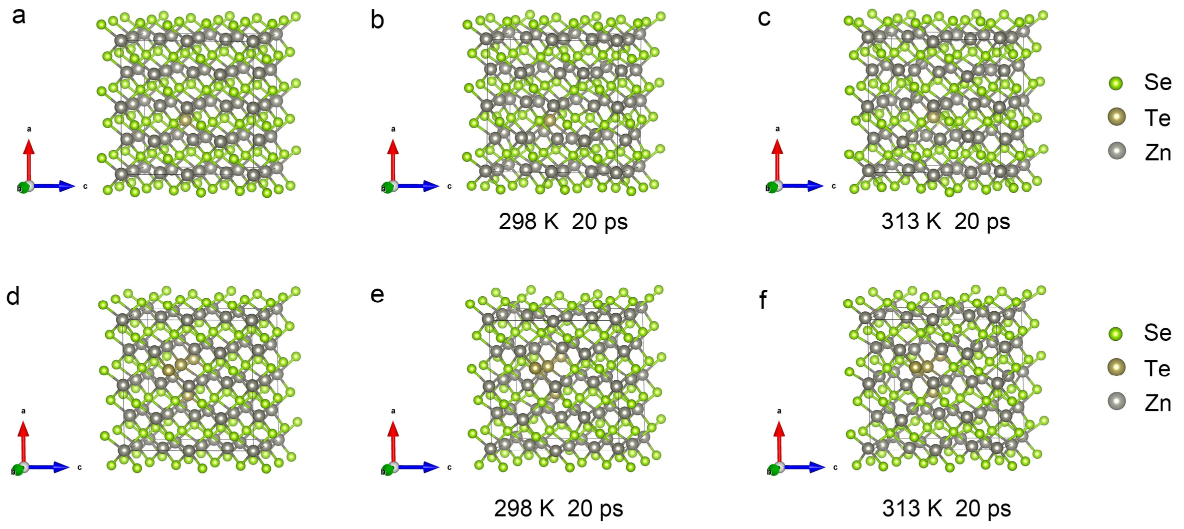

Supplementary Fig. 7 The models of **a** sub 1 after 20 ps **b** at 298 K and **c** at 313 K.

The models of **d** sub 3 after 20 ps **e** at 298 K and **f** at 313 K.

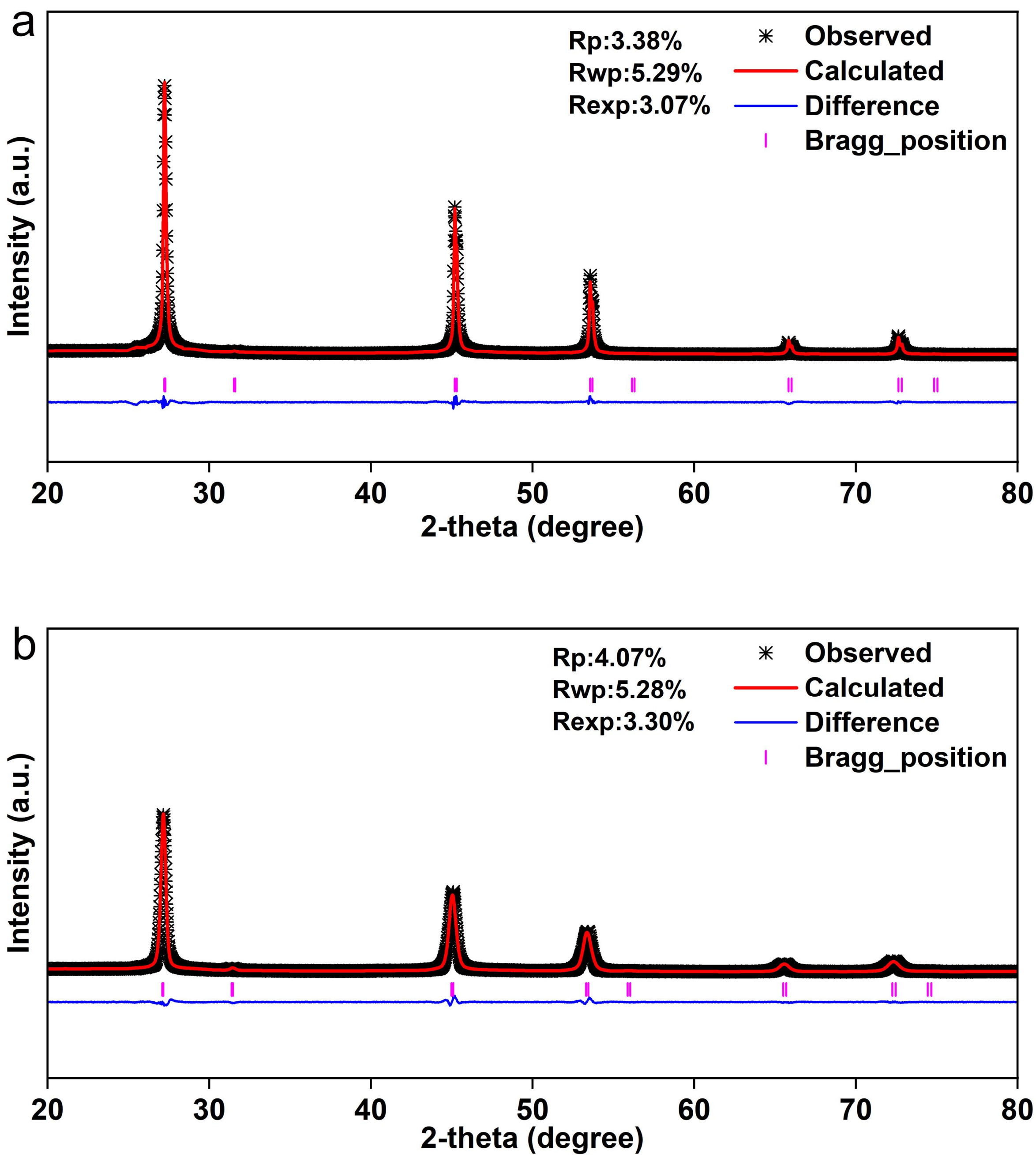

Supplementary Fig. 8 Rietveld refinement for powder XRD patterns of **a** ZnSe@C and **b** $ZnSe_{0.7}Te_{0.3}$@C materials.

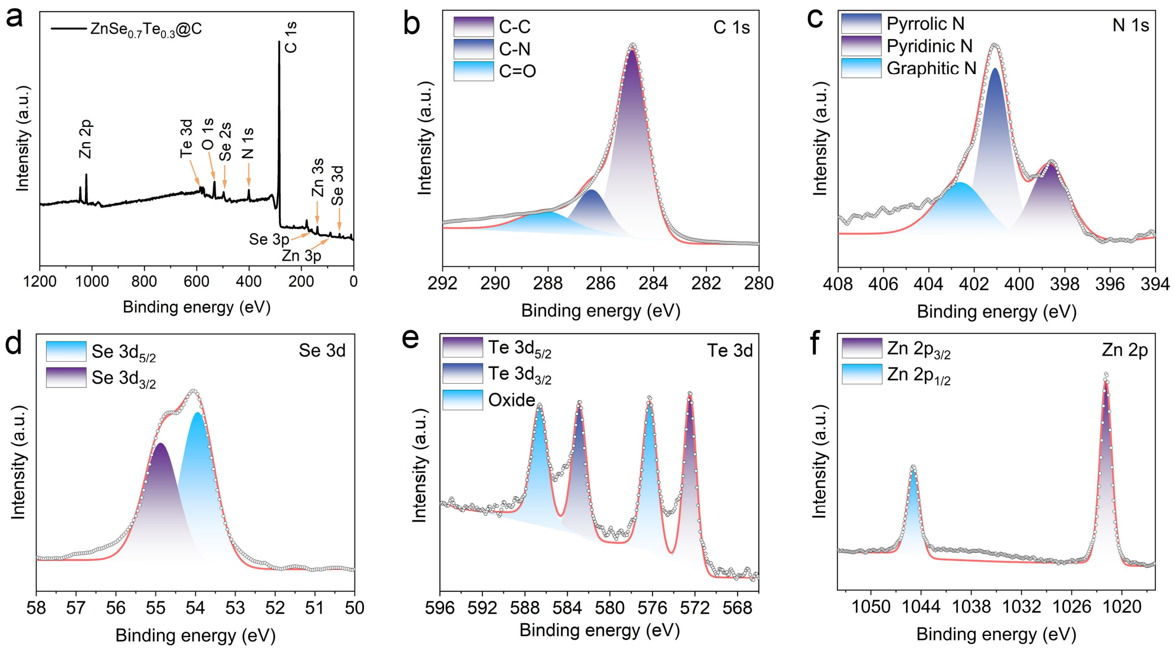

Supplementary Fig. 9 **a** The survey XPS spectrum of the $ZnSe_{0.7}Te_{0.3}$@C. High-resolution XPS spectra of $ZnSe_{0.7}Te_{0.3}$@C for **b** C 1*s*, **c** N 1*s*, **d** Se 3*d*, **e** Te 3*d*, **f** Zn 2*p*.

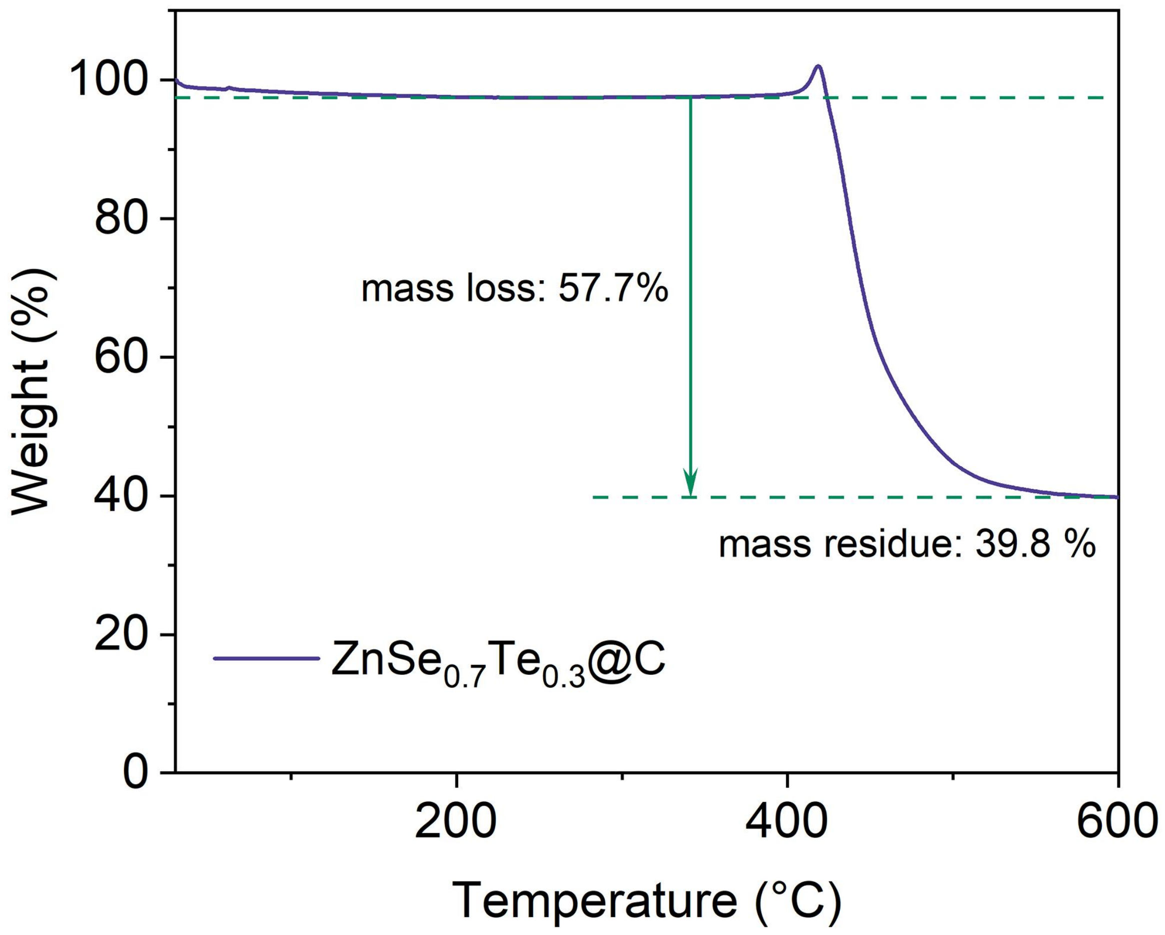

Supplementary Fig. 10 TGA curve of the $ZnSe_{0.7}Te_{0.3}$@C nanocomposites.

Note: The thermogravimetric analysis test of $ZnSe_{0.7}Te_{0.3}$@C nanocomposites was carried out in an air atmosphere. After heating, $ZnSe_{0.7}Te_{0.3}$@C decomposed into ZnO, TeO_2, SeO_2, and CO_2, with the final solid product of ZnO and TeO_2[1,2]. Therefore, the content of carbon in $ZnSe_{0.7}Te_{0.3}$@C can be calculated as follows:

$$P_{ZnSe_{0.7}Te_{0.3}} = \frac{m_{residue} \times M_{ZnSe_{0.7}Te_{0.3}}}{M_{ZnO} + 0.3M_{TeO_2}} \qquad (1)$$

$$P_{carbon} = 97.5\% - P_{ZnSe_{0.7}Te_{0.3}} \qquad (2)$$

where P means percent, $m_{residue}$=39.8%, M_{TeO2} =159.6 g/mol, M_{ZnO} = 81.4 g/mol, $M_{ZnSe0.7Te0.3}$ = 158.9 g/mol.

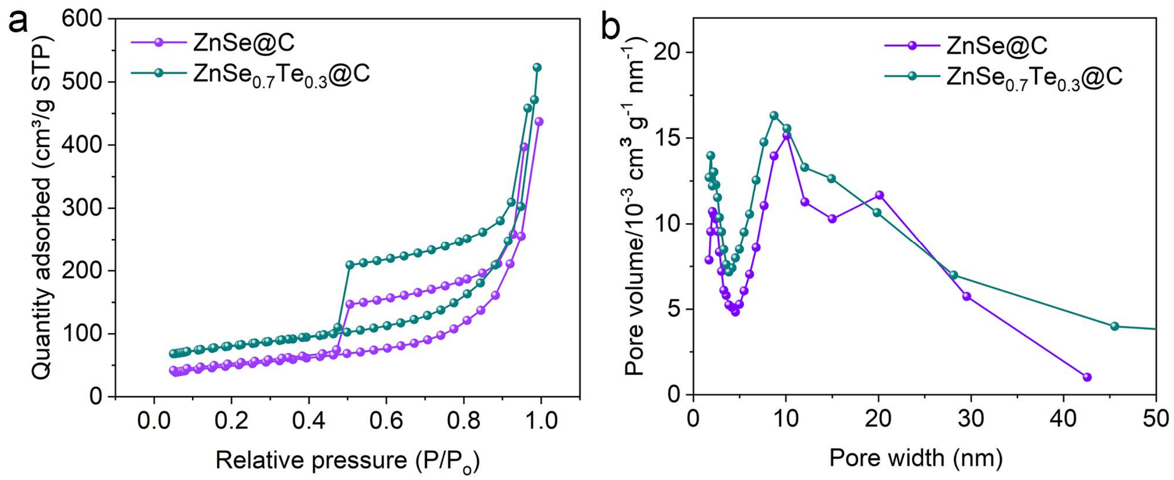

Supplementary Fig. 11 a Nitrogen adsorption/desorption isotherms and **b** the pore size distribution curves of ZnSe@C and $ZnSe_{0.7}Te_{0.3}$@C.

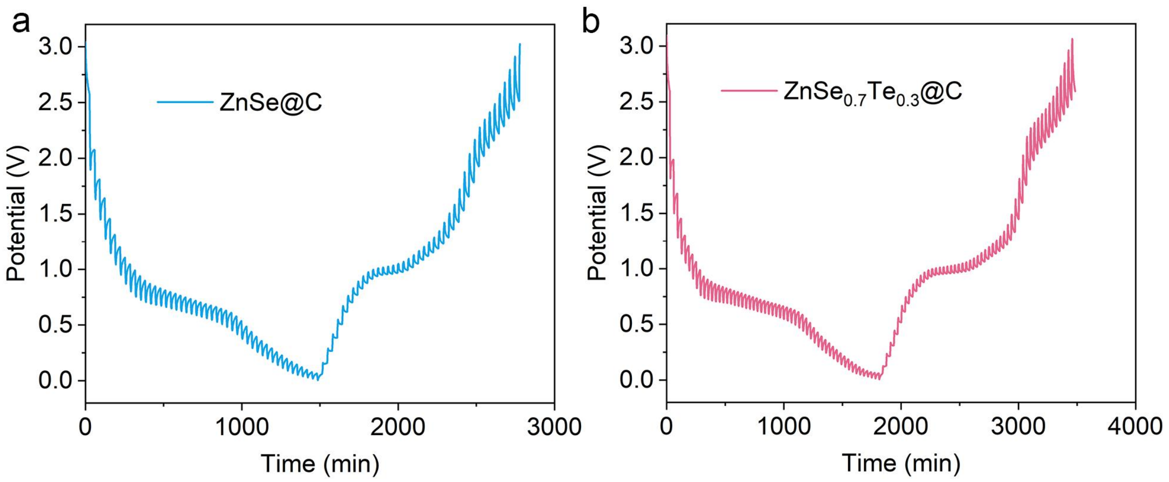

Supplementary Fig. 12 GITT curves of **a** ZnSe@C and **b** $ZnSe_{0.7}Te_{0.3}$@C.

Note: According to Fick's second law, the diffusion coefficient of sodium ions (D_{Na+}) is calculated by the GITT curve, and the formula is as follows[3]:

$$D_{\mathrm{Na^+}} = \frac{4}{\pi\tau}\left(\frac{m_{\mathrm{B}}V_{\mathrm{M}}}{M_{\mathrm{B}}S}\right)^2\left(\frac{\Delta E_{\mathrm{s}}}{\Delta E_{\mathrm{t}}}\right)^2 \qquad (3)$$

Where τ is the duration of the pulse current, m_B is the active material mass, M_B is the molar mass of active material, V_M is the molar volume of the active material, S is the contact area between the electrode and the electrolyte, ΔE_s is the steady-state voltage change caused by the pulse, and ΔE_t is the voltage change of constant current charge and discharge.

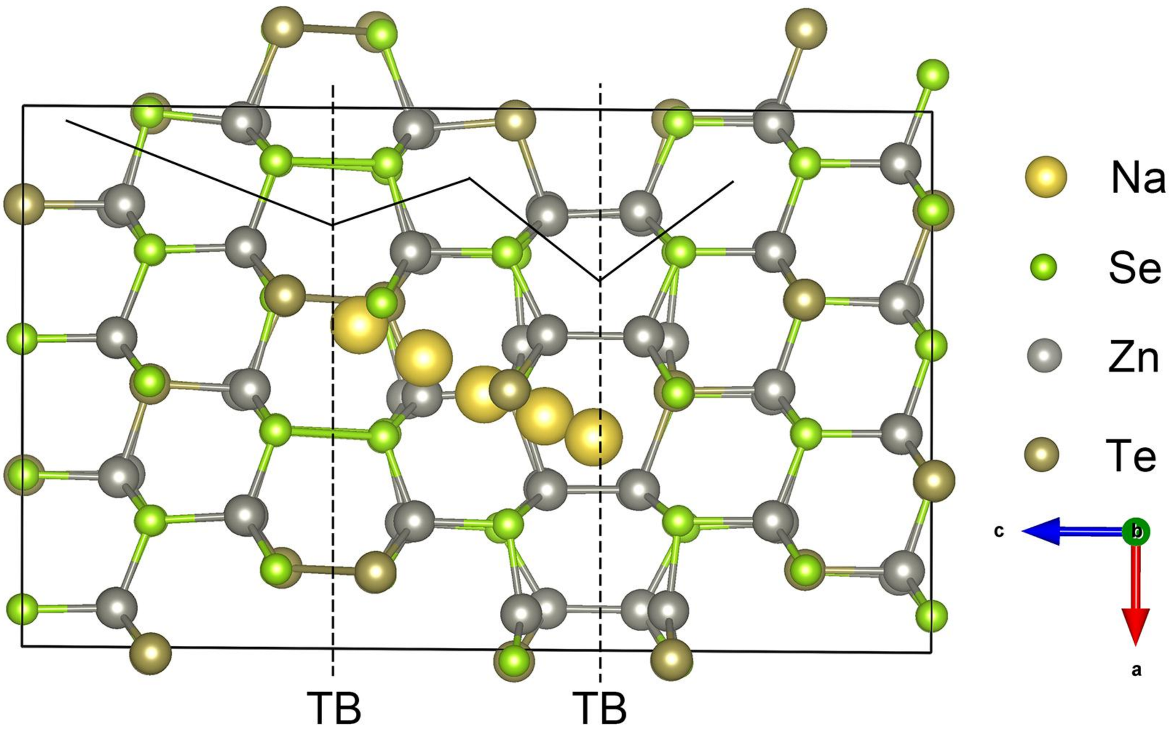

Supplementary Fig. 13 Model of $ZnSe_{0.7}Te_{0.3}$ with two TBs.

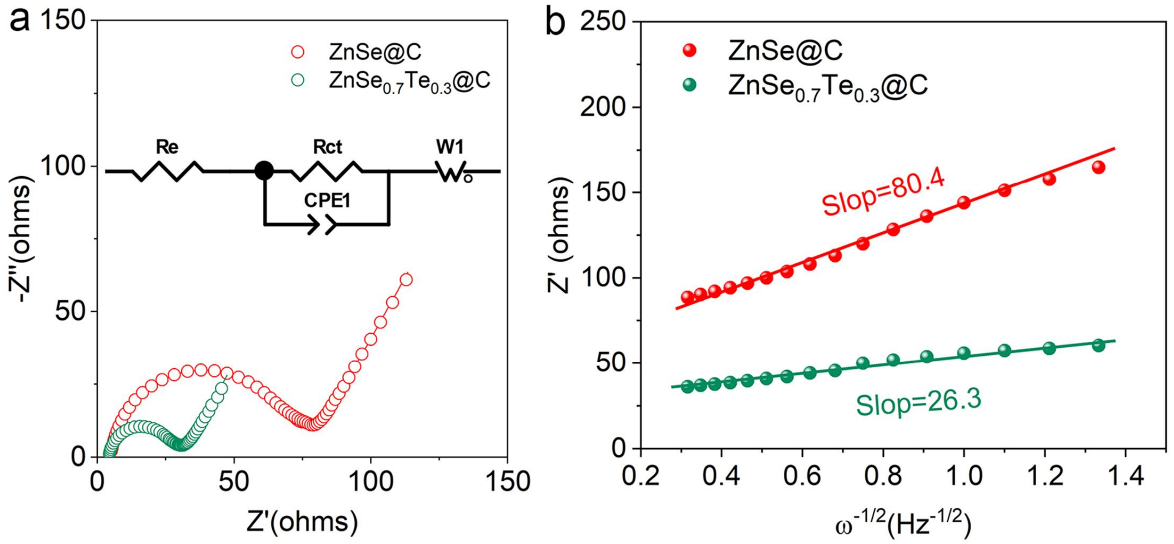

Supplementary Fig. 14 a EIS spectra and equivalent circuit model of ZnSe@C and $ZnSe_{0.7}Te_{0.3}$@C (line: fitted data; dot: pristine data). **b** The corresponding Z'-$\omega^{-1/2}$ plots.

Note: The sodium ion diffusion coefficient (D_{Na+}) and Warburg coefficient σ can be estimated using the following formula[4].

$$Z' = R_e + R_{ct} + \sigma\omega^{-1/2} \quad (4)$$

$$D = R^2T^2/2n^4F^4C^2\sigma^2A^2 \quad (5)$$

Where T is the Kelvin temperature (T= 298 K), R is the gas constant (R= 8.314 J K^{-1} mol^{-1}), A is the electrode area, F is Faraday's constant (F = 96500 C mol^{-1}), n is the number of electrons transferred during the reaction, and C is the Na^+ bulk phase concentration.

All Nyquist plots consist of oblique lines in the low frequency region and flat semicircles in the high frequency region, as shown in Supplementary Fig. 14a. In the fitted equivalent circuit, R_e represents the ohmic resistance in the cell system, which is related to the semicircular intercept of the Z' axis. CPE1 represents a double layer

between the electrode and the electrolyte. R_{ct} is the charge transfer resistance, which represents the resistance of charge transfer at the interface between the electrode and the electrolyte. The smaller the diameter of the semicircle, the lower the charge transfer resistance (R_{ct}), the higher its conductivity. As shown in Supplementary Fig. 14b, the slope of the oblique line represents σ, related to the diffusion of sodium ions (D_{Na+}). The lower the slope, the faster the sodium diffusion rate (D_{Na+}), as shown in Supplementary Fig. 14b.

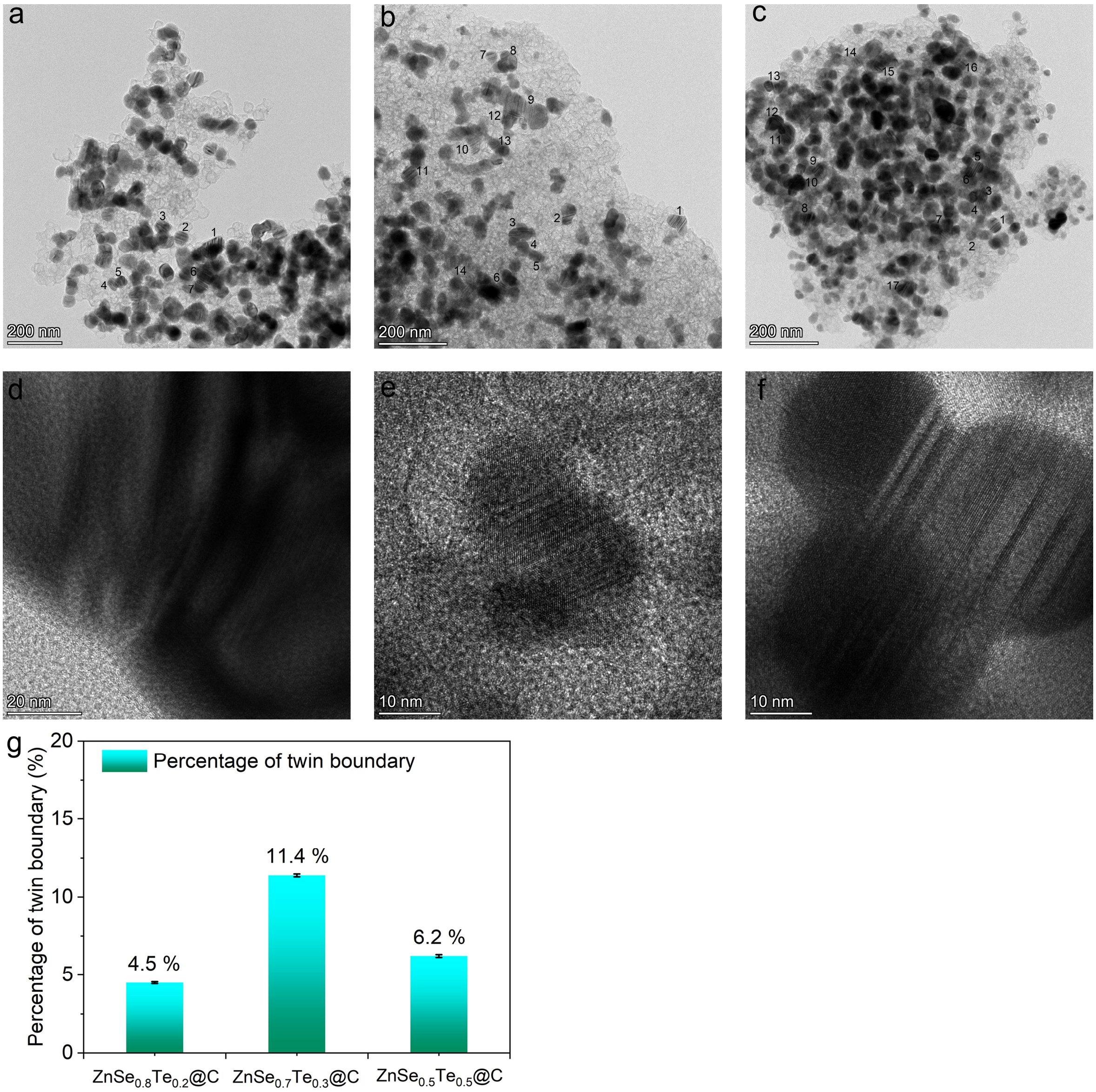

Supplementary Fig. 15 TEM and HRTEM images of **a, d** $ZnSe_{0.8}Te_{0.2}$@C, **b, e** $ZnSe_{0.7}Te_{0.3}$@C and **c, f** $ZnSe_{0.5}Te_{0.5}$@C. **g** Percentage of the number of TBs and error bars in $ZnSe_{0.8}Te_{0.2}$@C, $ZnSe_{0.7}Te_{0.3}$@C and $ZnSe_{0.5}Te_{0.5}$@C.

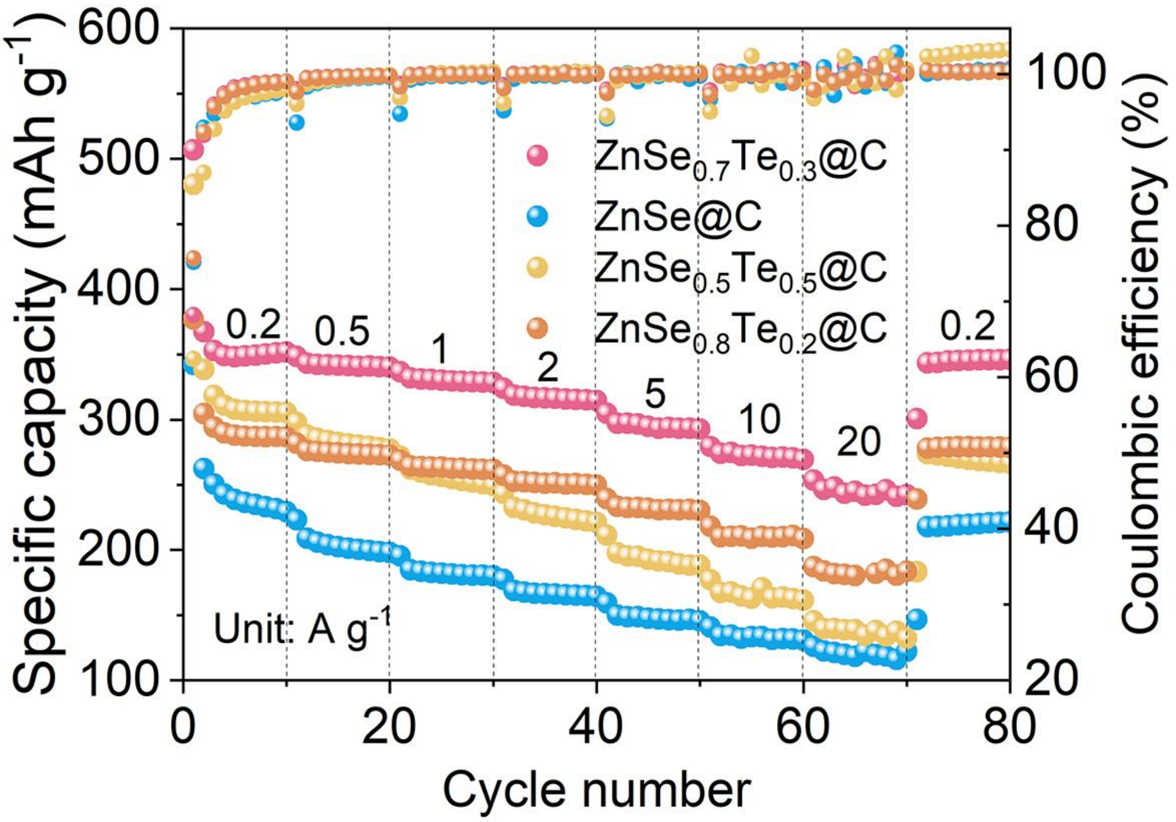

Supplementary Fig. 16 The rate performance of other batteries for the $ZnSe_{0.7}Te_{0.3}$@C, ZnSe@C, $ZnSe_{0.5}Te_{0.5}$@C and $ZnSe_{0.8}Te_{0.2}$@C. Test temperature: 25(±0.5)°C. Type of electrolyte: 1 M $NaPF_6$ in dimethoxyethane.

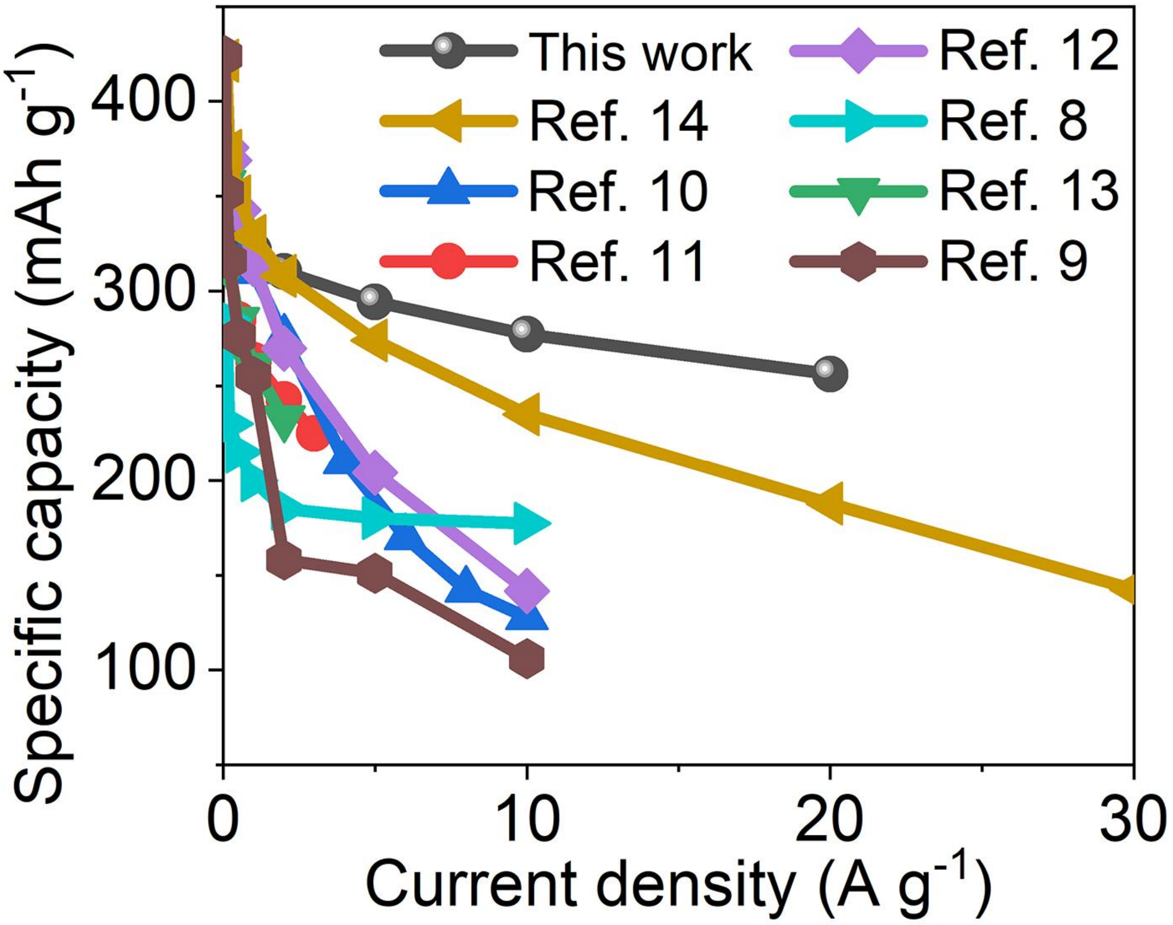

Supplementary Fig. 17 Comparison of the rate performance with those previously reported zinc–based selenides and tellurides[8-14].

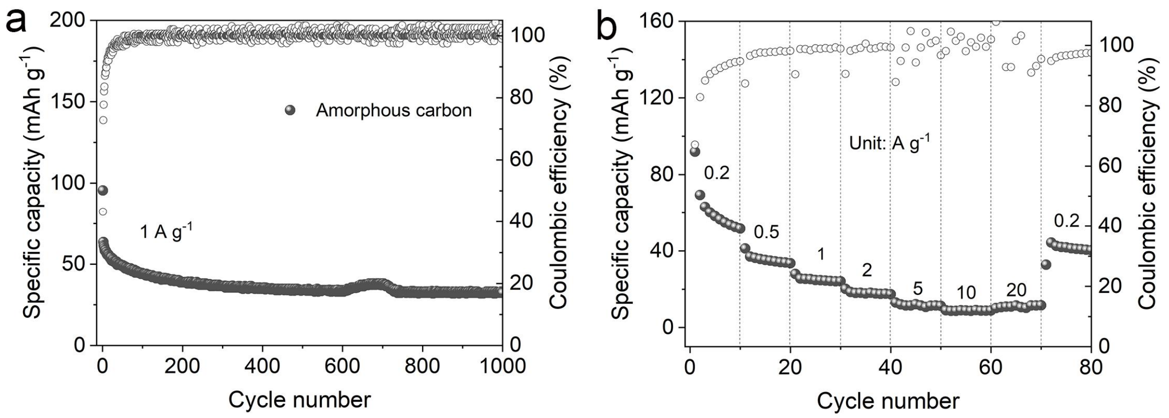

Supplementary Fig. 18 a Cycle performance and **b** rate performance of amorphous carbon.

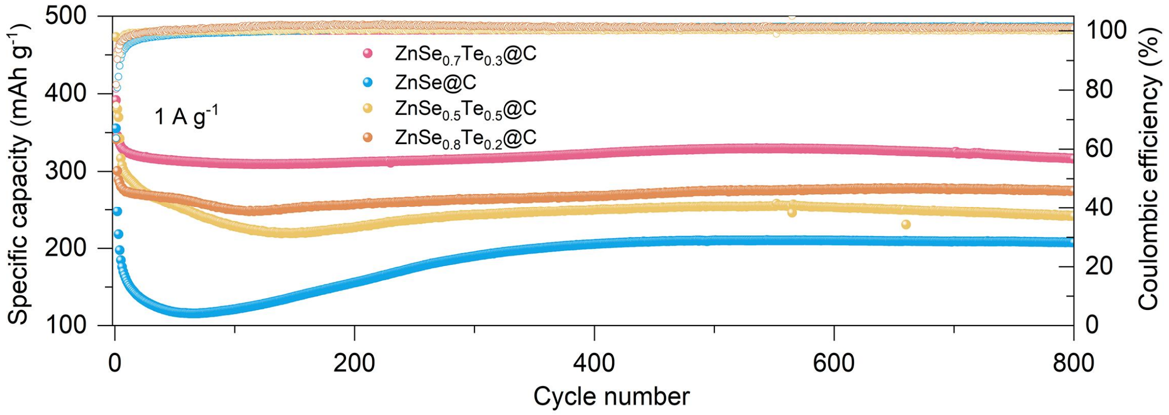

Supplementary Fig. 19 The cycle performance of other battery at the current density of 1 A g^{-1} for $ZnSe_{0.7}Te_{0.3}$@C, ZnSe@C, $ZnSe_{0.5}Te_{0.5}$@C and $ZnSe_{0.8}Te_{0.2}$@C. Test temperature: 25(±0.5)°C. Type of electrolyte: 1 M $NaPF_6$ in dimethoxyethane.

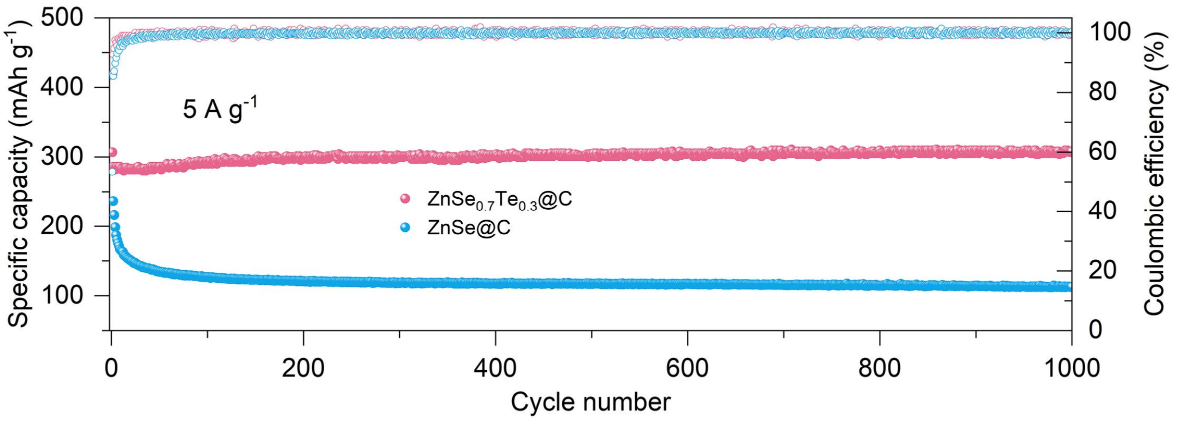

Supplementary Fig. 20 The cycle performance of other batteries at the current density of 5 A g^{-1} for $ZnSe_{0.7}Te_{0.3}$@C and ZnSe@C. Test temperature: 25(±0.5)°C. Type of electrolyte: 1 M $NaPF_6$ in dimethoxyethane.

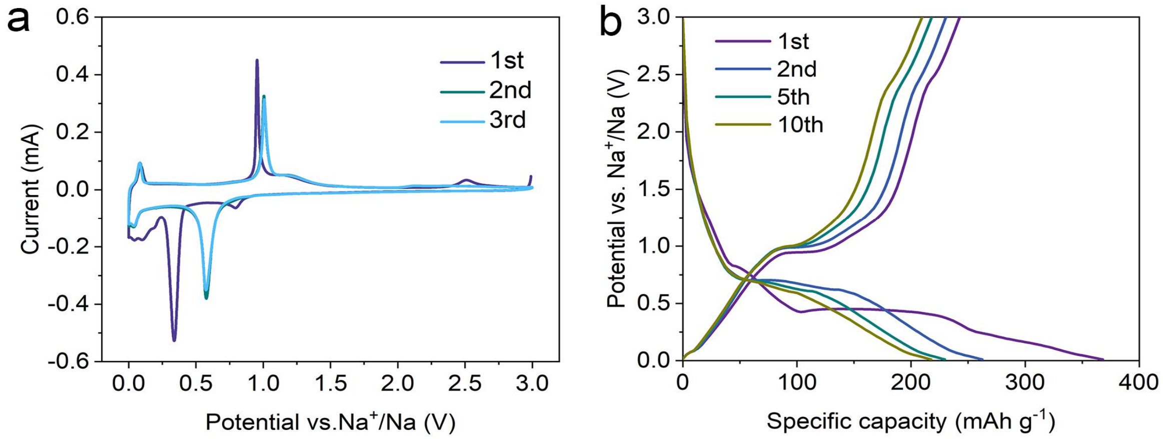

Supplementary Fig. 21 a CV curves and **b** galvanostatic charge and discharge curves at a current density of 0.2 A g^{-1} of ZnSe@C.

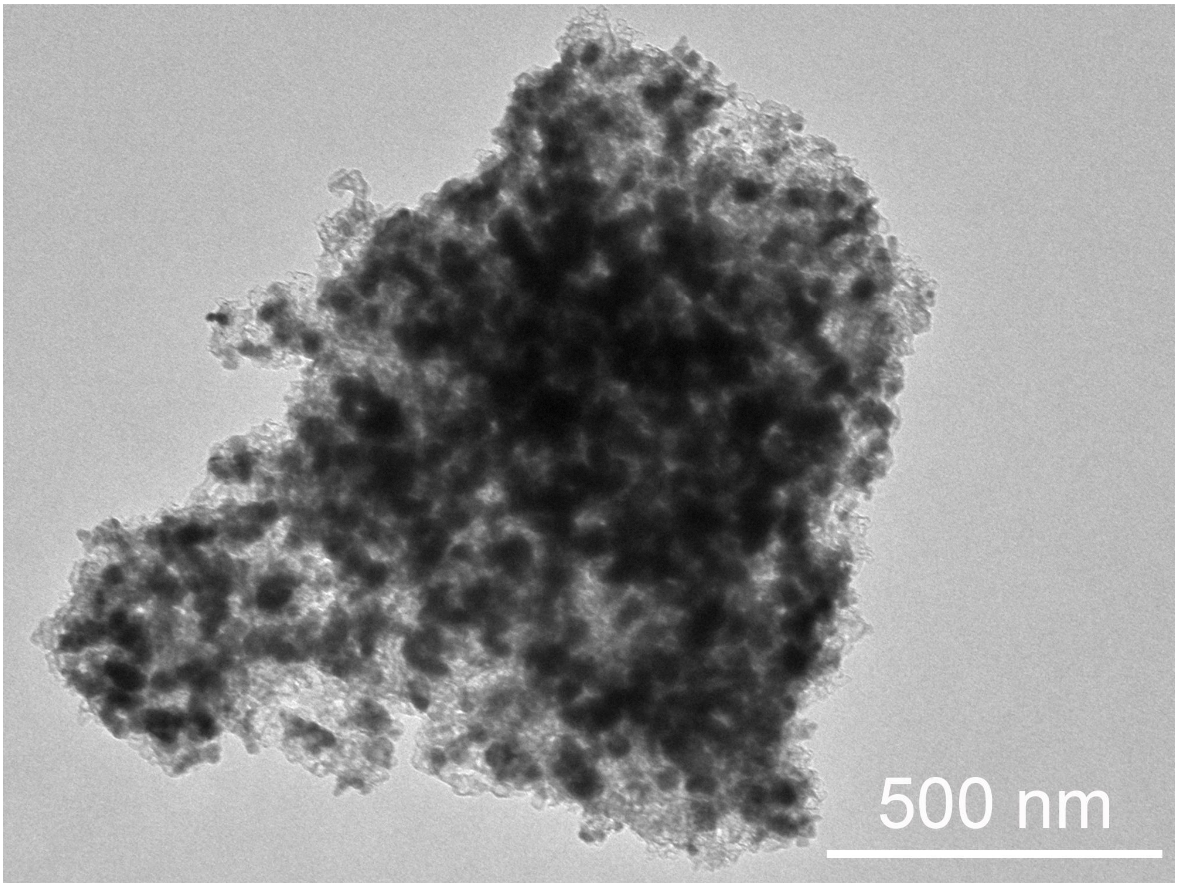

Supplementary Fig. 22 TEM image of ZnSe@C/Te.

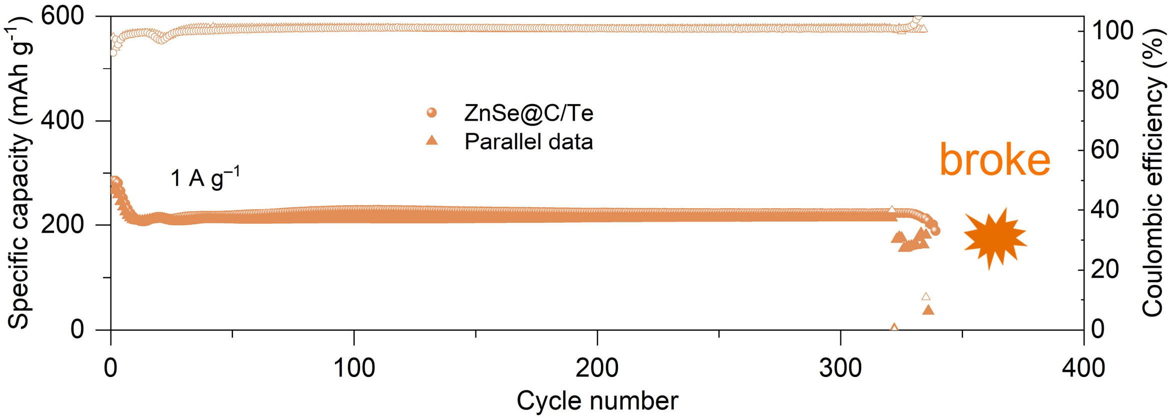

Supplementary Fig. 23 Long–term cycling of ZnSe@C/Te and parallel battery at the density of 1 A g^{-1}.

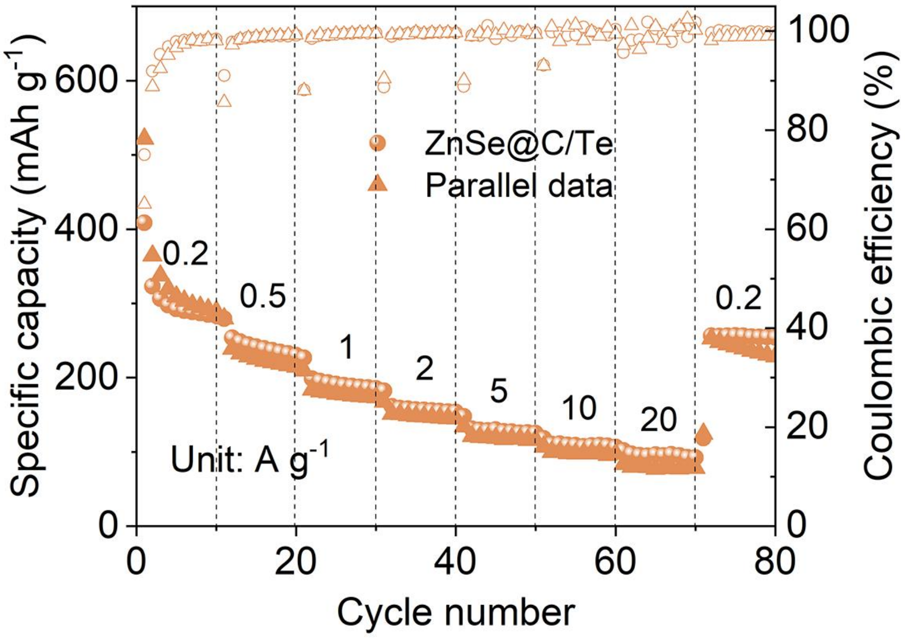

Supplementary Fig. 24 Rate performance of ZnSe@C/Te and parallel battery.

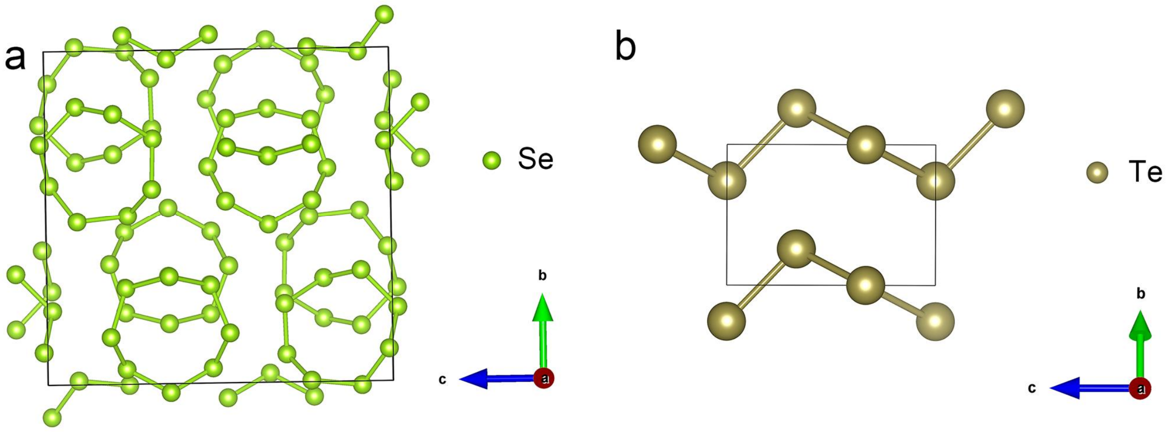

Supplementary Fig. 25 Solid structure models of **a** Se and **b** Te after structure optimization.

Supplementary Table 1. Atomic parameters of Rietveld refinement for powder XRD pattern of ZnSe@C.

Atom	x	y	z	Occupancy
Zn1	0.00000	0.00000	0.00000	0.042
Se1	0.25000	0.25000	0.25000	0.042

Supplementary Table 2. Atomic parameters of Rietveld refinement for powder XRD pattern of $ZnSe_{0.7}Te_{0.3}$@C.

Atom	x	y	z	Occupancy
Zn1	0.00000	0.00000	0.00000	0.042
Se1	0.25000	0.25000	0.25000	0.029
Te1	0.25000	0.25000	0.25000	0.013

Supplementary Table 3. Cell parameters of Rietveld refinement for powder XRD patterns of ZnSe@C and $ZnSe_{0.7}Te_{0.3}$@C.

Sample	a/b/c (Å)	α/β/γ (º)	V(Å^3)
ZnSe@C	5.66911	90.00000	182.1984
$ZnSe_{0.7}Te_{0.3}$@C	5.69440	90.00000	184.6477

Supplementary Table 4. The crystallite size of ZnSe@C and $ZnSe_{0.7}Te_{0.3}$@C from XRD characterization according to Scherrer's relation.

ZnSe@C		$ZnSe_{0.7}Te_{0.3}$@C	
2θ	particle size (nm)	2θ	particle size (nm)
27.21	43.5	27.15	25.7
45.24	42.3	45.04	18.5
53.60	44.3	53.36	16.3
65.93	44.6	65.56	14.9
72.74	42.5	72.28	13.8

Note: Scherrer's formula is used to calculate the size of particles[5].

$$D = \frac{K\lambda}{\beta \cos\theta} \quad (6)$$

where D represents the size of the grain, K is constant, λ is the X-ray wavelength, β is the full width of the diffraction peak at half maxima, θ represents the diffraction angle.

Supplementary Table 5. Equivalent circuit fitting value of ZnSe@C and $ZnSe_{0.7}Te_{0.3}$@C samples.

Sample	ZnSe@C	$ZnSe_{0.7}Te_{0.3}$@C
R_e	4.61	3.51
R_{ct}	68.26	21.83

Supplementary Table 6. Energy-dispersive X-ray (EDX) analysis of C, N, O, Zn, Se and Te atomic percentages for $ZnSe_{0.8}Te_{0.2}$@C, $ZnSe_{0.7}Te_{0.3}$@C and $ZnSe_{0.5}Te_{0.5}$@C.

Atomic %	$ZnSe_{0.8}Te_{0.2}$@C	$ZnSe_{0.7}Te_{0.3}$@C	$ZnSe_{0.5}Te_{0.5}$@C
C	84.49	81.75	82.30
N	3.41	3.11	2.99
O	1.26	2.02	3.52
Zn	6.18	7.05	5.90
Se	3.73	4.13	2.57
Te	0.93	1.93	2.72

Note 1: Calculations of elastic stiffness constant for the structural models of sub 1, sub 3 and int.

The sub 1 model with one Te atom replacing one Se atom belongs to the cubic crystal system and has only three independent constants, C_{11}, C_{12} and C_{44}.

$$C = \begin{pmatrix} C_{11} & C_{12} & C_{12} & 0 & 0 & 0 \\ C_{12} & C_{11} & C_{12} & 0 & 0 & 0 \\ C_{12} & C_{12} & C_{11} & 0 & 0 & 0 \\ 0 & 0 & 0 & C_{44} & 0 & 0 \\ 0 & 0 & 0 & 0 & C_{44} & 0 \\ 0 & 0 & 0 & 0 & 0 & C_{44} \end{pmatrix} \quad (7)$$

According to the mechanical stability criteria[6,7], mechanical stability requires the elastic constants of cubic crystal systems to meet the following requirements:

$$C_{44} > 0, \quad C_{11} - C_{12} > 0, \quad C_{11} + 2C_{12} > 0 \quad (8)$$

The elastic constant of sub 1 model can be obtained by theoretical calculation as follows:

$$C_{\mathrm{sub\ 1}} = \begin{pmatrix} 92.196 & 56.159 & 56.159 & 0 & 0 & 0 \\ 56.159 & 92.196 & 56.159 & 0 & 0 & 0 \\ 56.159 & 56.159 & 92.196 & 0 & 0 & 0 \\ 0 & 0 & 0 & 39.666 & 0 & 0 \\ 0 & 0 & 0 & 0 & 39.666 & 0 \\ 0 & 0 & 0 & 0 & 0 & 39.666 \end{pmatrix} \quad (9)$$

The elastic constants of sub 1 model satisfy the criteria in formula (8), so sub 1 model is mechanically stable.

The sub 3 model with three Te atoms replacing three Se atoms belongs to the monoclinic crystal system, and has 13 independent elastic constants.

$$C=\begin{pmatrix} C_{11} & C_{12} & C_{13} & 0 & C_{15} & 0 \\ C_{12} & C_{22} & C_{23} & 0 & C_{25} & 0 \\ C_{13} & C_{23} & C_{33} & 0 & C_{35} & 0 \\ 0 & 0 & 0 & C_{44} & 0 & C_{46} \\ C_{15} & C_{25} & C_{35} & 0 & C_{55} & 0 \\ 0 & 0 & 0 & C_{46} & 0 & C_{66} \end{pmatrix} \tag{10}$$

Similarly, mechanical stability requires that the elastic constants of monoclinic crystal systems meet the following requirements:

$$C_{11} > 0,\quad C_{11}C_{22} - C_{12}^2 > 0,\quad C_{11}C_{22}C_{33} + 2C_{12}C_{13}C_{23} - C_{11}C_{23}^2 - C_{22}C_{13}^2 - C_{33}C_{12}^2 > 0,$$
$$C_{44} > 0,\quad C_{11}(C_{22}C_{33}C_{55} + 2C_{23}C_{25}C_{35} - C_{33}C_{25}^2 - C_{22}C_{35}^2 - C_{55}C_{23}^2) - C_{12}(C_{12}C_{33}C_{55} + C_{23}C_{15}C_{35} + C_{13}C_{25}C_{35} - C_{33}C_{15}C_{25} - C_{12}C_{35}^2 - C_{13}C_{23}C_{55}) + C_{13}(C_{12}C_{23}C_{55} + C_{22}C_{35}C_{15} + C_{13}C_{25}^2 - C_{23}C_{15}C_{25} - C_{12}C_{25}C_{35} - C_{22}C_{13}C_{55}) - C_{15}(C_{12}C_{23}C_{35} + C_{22}C_{33}C_{15} + C_{13}C_{23}C_{25} - C_{15}C_{23}^2 - C_{12}C_{33}C_{25} - C_{22}C_{13}C_{35}) > 0,\quad C_{44}C_{66} - C_{46}^2 > 0, \tag{11}$$

The elastic constants of sub 3 model can be obtained by theoretical calculation as follows:

$$C_{\text{sub 3}}=\begin{pmatrix} 91.476 & 54.352 & 54.458 & 0 & -0.310 & 0 \\ 54.352 & 91.439 & 54.476 & 0 & 0.172 & 0 \\ 54.458 & 54.476 & 90.803 & 0 & 0.350 & 0 \\ 0 & 0 & 0 & 39.089 & 0 & 0.697 \\ -0.310 & 0.172 & 0.350 & 0 & 39.135 & 0 \\ 0 & 0 & 0 & 0.697 & 0 & 39.550 \end{pmatrix} \tag{12}$$

The elastic constants of sub 3 model satisfy the criteria in formula (11), so sub 3 model is mechanically stable.

The int model with one Te atom occupying interstitial position belongs to the triclinic crystal system, and has 21 independent elastic constants.

$$C = \begin{pmatrix} C_{11} & C_{12} & C_{13} & C_{14} & C_{15} & C_{16} \\ C_{12} & C_{22} & C_{23} & C_{24} & C_{25} & C_{26} \\ C_{13} & C_{23} & C_{33} & C_{34} & C_{35} & C_{36} \\ C_{14} & C_{24} & C_{34} & C_{44} & C_{45} & C_{46} \\ C_{15} & C_{25} & C_{35} & C_{45} & C_{55} & C_{56} \\ C_{16} & C_{26} & C_{36} & C_{46} & C_{56} & C_{66} \end{pmatrix} \quad (13)$$

Similarly, mechanical stability requires that the elastic constants of triclinic crystal systems meet the following requirements:

$C_{11} > 0,\quad C_{11}C_{22} - C_{12}^2 > 0,\quad C_{11}C_{22}C_{33} + 2C_{12}C_{13}C_{23} - C_{11}C_{23}^2 - C_{22}C_{13}^2 - C_{33}C_{12}^2 > 0,$

$C_{11}(C_{22}C_{33}C_{44} + 2C_{23}C_{24}C_{34} - C_{33}C_{24}^2 - C_{22}C_{34}^2 - C_{44}C_{23}^2) - C_{12}(C_{12}C_{33}C_{44} + C_{23}C_{14}C_{34} + C_{13}C_{24}C_{34} - C_{33}C_{14}C_{24} - C_{12}C_{34}^2 - C_{13}C_{23}C_{44}) + C_{13}(C_{12}C_{23}C_{44} + C_{22}C_{14}C_{34} + C_{13}C_{24}^2 - C_{23}C_{14}C_{24} - C_{12}C_{24}C_{34} - C_{22}C_{13}C_{44}) - C_{14}(C_{12}C_{23}C_{34} + C_{22}C_{33}C_{14} + C_{13}C_{23}C_{24} - C_{14}C_{23}^2 - C_{12}C_{33}C_{24} - C_{22}C_{13}C_{34}) > 0,$

$C_{11}(a - b + c - d) - C_{12}(e - f + g - h) + C_{13}(i - j + k - l) - C_{14}(m - n + o - p) + C_{15}(q - r + s - t) > 0,$

$$\sum_{i=1}^{20} A_i B_i > 0 \quad (14)$$

where $a = C_{22}(C_{33}C_{44}C_{55} + 2C_{34}C_{35}C_{45} - C_{44}C_{35}^2 - C_{33}C_{45}^2 - C_{55}C_{34}^2)$,

$b = C_{23}(C_{23}C_{44}C_{55} + C_{34}C_{25}C_{45} + C_{24}C_{35}C_{45} - C_{44}C_{25}C_{35} - C_{23}C_{45}^2 - C_{24}C_{34}C_{55})$,

$c = C_{24}(C_{23}C_{34}C_{55} + C_{33}C_{45}C_{25} + C_{24}C_{35}^2 - C_{34}C_{25}C_{35} - C_{23}C_{35}C_{45} - C_{33}C_{24}C_{55})$,

$d = C_{25}(C_{23}C_{34}C_{45} + C_{33}C_{44}C_{25} + C_{24}C_{34}C_{35} - C_{34}^2C_{25} - C_{23}C_{44}C_{35} - C_{33}C_{24}C_{45})$,

$e = C_{12}(C_{33}C_{44}C_{55} + 2C_{34}C_{35}C_{45} - C_{44}C_{35}^2 - C_{33}C_{45}^2 - C_{55}C_{34}^2)$,

$f = C_{23}(C_{13}C_{44}C_{55} + C_{34}C_{15}C_{45} + C_{14}C_{35}C_{45} - C_{44}C_{15}C_{35} - C_{13}C_{45}^2 - C_{14}C_{34}C_{55})$,

$g = C_{24}(C_{13}C_{34}C_{55} + C_{33}C_{45}C_{15} + C_{14}C_{35}^2 - C_{34}C_{15}C_{35} - C_{13}C_{35}C_{45} - C_{33}C_{14}C_{55})$,

$h = C_{25}(C_{13}C_{34}C_{45} + C_{33}C_{44}C_{15} + C_{14}C_{34}C_{35} - C_{34}^2C_{15} - C_{13}C_{44}C_{35} - C_{33}C_{14}C_{45})$,

$i = C_{12}(C_{23}C_{44}C_{55} + C_{34}C_{25}C_{45} + C_{24}C_{35}C_{45} - C_{44}C_{25}C_{35} - C_{23}C_{45}^2 - C_{24}C_{34}C_{55})$,

$j = C_{22}(C_{13}C_{44}C_{55} + C_{34}C_{15}C_{45} + C_{14}C_{35}C_{45} - C_{44}C_{15}C_{35} - C_{13}C_{45}^2 - C_{14}C_{34}C_{55})$,

$k = C_{24}(C_{13}C_{24}C_{55} + C_{23}C_{15}C_{45} + C_{14}C_{25}C_{35} - C_{24}C_{15}C_{35} - C_{13}C_{25}C_{45} - C_{23}C_{14}C_{55})$,

$l = C_{25}(C_{13}C_{24}C_{45} + C_{23}C_{44}C_{15} + C_{14}C_{34}C_{25} - C_{24}C_{34}C_{15} - C_{13}C_{44}C_{25} - C_{23}C_{14}C_{45})$,

$m = C_{12}(C_{23}C_{34}C_{55} + C_{33}C_{25}C_{45} + C_{24}C_{35}^2 - C_{34}C_{25}C_{35} - C_{23}C_{35}C_{45} - C_{33}C_{24}C_{55})$,

$n = C_{22}(C_{13}C_{34}C_{55} + C_{33}C_{15}C_{45} + C_{14}C_{35}^2 - C_{34}C_{15}C_{35} - C_{13}C_{35}C_{45} - C_{33}C_{14}C_{55})$,

$\mathrm{o} = C_{23}(C_{13}C_{24}C_{55} + C_{23}C_{45}C_{15} + C_{14}C_{25}C_{35} - C_{24}C_{15}C_{35} - C_{13}C_{25}C_{45} - C_{23}C_{14}C_{55})$,

$p = C_{25}(C_{13}C_{24}C_{35} + C_{23}C_{34}C_{15} + C_{33}C_{14}C_{25} - C_{33}C_{24}C_{15} - C_{13}C_{34}C_{25} - C_{23}C_{14}C_{35})$,

$q = C_{12}(C_{23}C_{34}C_{45} + C_{33}C_{44}C_{25} + C_{24}C_{34}C_{35} - C_{25}C_{34}^2 - C_{23}C_{44}C_{35} - C_{24}C_{33}C_{45})$,

$r = C_{22}(C_{13}C_{34}C_{45} + C_{33}C_{44}C_{15} + C_{14}C_{34}C_{35} - C_{15}C_{34}^2 - C_{13}C_{44}C_{35} - C_{33}C_{14}C_{45})$,

$s = C_{23}(C_{13}C_{24}C_{45} + C_{23}C_{44}C_{15} + C_{14}C_{25}C_{34} - C_{34}C_{24}C_{15} - C_{13}C_{25}C_{44} - C_{14}C_{23}C_{45})$,

$t = C_{24}(C_{13}C_{24}C_{35} + C_{23}C_{34}C_{15} + C_{14}C_{25}C_{33} - C_{33}C_{24}C_{15} - C_{13}C_{34}C_{25} - C_{23}C_{14}C_{35})$,

$B_1 = C_{14}C_{25}C_{36} + C_{24}C_{35}C_{16} + C_{34}C_{15}C_{26} - C_{16}C_{25}C_{34} - C_{14}C_{35}C_{26} - C_{24}C_{15}C_{36}$,

$B_2 = C_{14}C_{25}C_{46} + C_{24}C_{45}C_{16} + C_{44}C_{15}C_{26} - C_{44}C_{25}C_{16} - C_{14}C_{45}C_{26} - C_{24}C_{15}C_{46}$,

$B_3 = C_{14}C_{25}C_{56} + C_{24}C_{55}C_{16} + C_{15}C_{45}C_{26} - C_{25}C_{45}C_{16} - C_{14}C_{55}C_{26} - C_{24}C_{15}C_{56}$,

$B_4 = C_{14}C_{25}C_{66} + C_{24}C_{56}C_{16} + C_{15}C_{26}C_{46} - C_{16}C_{25}C_{46} - C_{15}C_{24}C_{66} - C_{14}C_{26}C_{56}$,

$B_5 = C_{14}C_{35}C_{46} + C_{34}C_{45}C_{16} + C_{44}C_{15}C_{36} - C_{44}C_{35}C_{16} - C_{14}C_{45}C_{36} - C_{34}C_{15}C_{46}$,

$B_6 = C_{14}C_{35}C_{56} + C_{34}C_{55}C_{16} + C_{15}C_{36}C_{45} - C_{45}C_{35}C_{16} - C_{14}C_{55}C_{36} - C_{34}C_{15}C_{56}$,

$B_7 = C_{14}C_{35}C_{66} + C_{34}C_{56}C_{16} + C_{15}C_{36}C_{46} - C_{16}C_{35}C_{46} - C_{14}C_{36}C_{56} - C_{15}C_{34}C_{66}$,

$B_8 = C_{14}C_{45}C_{56} + C_{44}C_{55}C_{16} + C_{15}C_{45}C_{46} - C_{16}C_{45}^2 - C_{14}C_{46}C_{55} - C_{15}C_{44}C_{56}$,

$B_9 = C_{14}C_{45}C_{66} + C_{44}C_{56}C_{16} + C_{15}C_{46}^2 - C_{16}C_{45}C_{46} - C_{14}C_{46}C_{56} - C_{15}C_{44}C_{66}$,

$B_{10} = C_{14}C_{55}C_{66} + C_{45}C_{16}C_{56} + C_{15}C_{46}C_{56} - C_{55}C_{16}C_{46} - C_{14}C_{56}^2 - C_{15}C_{45}C_{66}$,

$B_{11} = C_{24}C_{35}C_{46} + C_{34}C_{45}C_{26} + C_{44}C_{25}C_{36} - C_{44}C_{35}C_{26} - C_{24}C_{45}C_{36} - C_{34}C_{25}C_{46}$,

$B_{12} = C_{24}C_{35}C_{56} + C_{34}C_{55}C_{26} + C_{25}C_{36}C_{45} - C_{35}C_{45}C_{26} - C_{24}C_{55}C_{36} - C_{34}C_{25}C_{56}$,

$B_{13} = C_{24}C_{35}C_{66} + C_{34}C_{26}C_{56} + C_{25}C_{36}C_{46} - C_{35}C_{26}C_{46} - C_{24}C_{36}C_{56} - C_{34}C_{25}C_{66}$,

$B_{14} = C_{24}C_{45}C_{56} + C_{44}C_{55}C_{26} + C_{25}C_{46}C_{45} - C_{45}^2C_{26} - C_{24}C_{55}C_{46} - C_{25}C_{44}C_{56}$,

$B_{15} = C_{24}C_{45}C_{66} + C_{44}C_{26}C_{56} + C_{25}C_{46}^2 - C_{45}C_{26}C_{46} - C_{24}C_{46}C_{56} - C_{44}C_{25}C_{66}$,

$B_{16} = C_{24}C_{55}C_{66} + C_{45}C_{56}C_{26} + C_{25}C_{56}C_{46} - C_{26}C_{55}C_{46} - C_{24}C_{56}^2 - C_{25}C_{45}C_{66}$,

$B_{17} = C_{34}C_{45}C_{56} + C_{44}C_{55}C_{36} + C_{35}C_{45}C_{46} - C_{36}C_{45}^2 - C_{44}C_{35}C_{56} - C_{34}C_{55}C_{46}$,

$B_{18} = C_{34}C_{45}C_{66} + C_{44}C_{36}C_{56} + C_{35}C_{46}^2 - C_{45}C_{36}C_{46} - C_{34}C_{46}C_{56} - C_{44}C_{35}C_{66}$,

$B_{19} = C_{34}C_{55}C_{66} + C_{45}C_{36}C_{56} + C_{35}C_{46}C_{56} - C_{55}C_{36}C_{46} - C_{34}C_{56}^2 - C_{35}C_{45}C_{66}$,

$B_{20} = C_{44}C_{55}C_{66} + C_{45}C_{56}C_{46} + C_{45}C_{46}C_{56} - C_{55}C_{46}^2 - C_{66}C_{45}^2 - C_{44}C_{56}^2$,

$A_1 = -C_{14}C_{25}C_{36} - C_{15}C_{34}C_{26} - C_{16}C_{24}C_{35} + C_{14}C_{35}C_{26} + C_{15}C_{24}C_{36} + C_{16}C_{34}C_{25}$,

$A_2 = C_{13}C_{25}C_{36} + C_{15}C_{33}C_{26} + C_{16}C_{23}C_{35} - C_{13}C_{26}C_{35} - C_{15}C_{23}C_{36} - C_{16}C_{25}C_{33}$,

$A_3 = -C_{13}C_{24}C_{36} - C_{14}C_{33}C_{26} - C_{16}C_{23}C_{34} + C_{13}C_{34}C_{26} + C_{14}C_{23}C_{36} + C_{16}C_{24}C_{33}$,

$A_4 = C_{13}C_{24}C_{35} + C_{14}C_{33}C_{25} + C_{15}C_{23}C_{34} - C_{13}C_{34}C_{25} - C_{14}C_{23}C_{35} - C_{15}C_{33}C_{24}$,

$A_5 = -C_{12}C_{25}C_{36} - C_{23}C_{15}C_{26} - C_{22}C_{35}C_{16} + C_{12}C_{35}C_{26} + C_{22}C_{15}C_{36} + C_{23}C_{25}C_{16}$,

$A_6 = C_{12}C_{24}C_{36} + C_{23}C_{14}C_{26} + C_{22}C_{34}C_{16} - C_{12}C_{34}C_{26} - C_{22}C_{14}C_{36} - C_{23}C_{24}C_{16}$,

$A_7 = -C_{12}C_{24}C_{35} - C_{23}C_{14}C_{25} - C_{22}C_{34}C_{15} + C_{12}C_{34}C_{25} + C_{22}C_{14}C_{35} + C_{23}C_{24}C_{15}$,

$A_8 = -C_{12}C_{23}C_{36} - C_{13}C_{23}C_{26} - C_{22}C_{33}C_{16} + C_{12}C_{33}C_{26} + C_{22}C_{13}C_{36} + C_{16}C_{23}^2$,

$A_9 = C_{12}C_{23}C_{35} + C_{13}C_{23}C_{25} + C_{22}C_{33}C_{15} - C_{12}C_{33}C_{25} - C_{22}C_{13}C_{35} - C_{15}C_{23}^2$,

$A_{10} = -C_{12}C_{23}C_{34} - C_{13}C_{23}C_{24} - C_{22}C_{33}C_{14} + C_{12}C_{33}C_{24} + C_{22}C_{13}C_{34} + C_{14}C_{23}^2$,

$A_{11} = C_{11}C_{25}C_{36} + C_{13}C_{15}C_{26} + C_{12}C_{35}C_{16} - C_{11}C_{35}C_{26} - C_{12}C_{15}C_{36} - C_{13}C_{25}C_{16}$,

$A_{12} = -\mathrm{C}_{11}\mathrm{C}_{24}\mathrm{C}_{36} - \mathrm{C}_{13}\mathrm{C}_{14}\mathrm{C}_{26} - \mathrm{C}_{12}\mathrm{C}_{16}\mathrm{C}_{34} + \mathrm{C}_{11}\mathrm{C}_{34}\mathrm{C}_{26} + \mathrm{C}_{12}\mathrm{C}_{14}\mathrm{C}_{36} + \mathrm{C}_{13}\mathrm{C}_{24}\mathrm{C}_{16}$,

$A_{13} = C_{11}C_{24}C_{35} + C_{13}C_{14}C_{25} + C_{12}C_{34}C_{15} - C_{12}C_{14}C_{35} - C_{13}C_{24}C_{15} - C_{11}C_{34}C_{25}$,

$A_{14} = C_{11}C_{23}C_{36} + C_{26}C_{13}^2 + C_{12}C_{33}C_{16} - C_{11}C_{33}C_{26} - C_{12}C_{13}C_{36} - C_{13}C_{23}C_{16}$,

$A_{15} = -C_{11}C_{23}C_{35} - C_{25}C_{13}^2 - C_{12}C_{33}C_{15} + C_{11}C_{33}C_{25} + C_{12}C_{13}C_{35} + C_{13}C_{23}C_{15}$,

$A_{16} = C_{11}C_{23}C_{34} + C_{24}C_{13}^2 + C_{12}C_{33}C_{14} - C_{11}C_{33}C_{24} - C_{12}C_{13}C_{34} - C_{13}C_{23}C_{14}$,

$A_{17} = -C_{11}C_{22}C_{36} - C_{12}C_{13}C_{26} - C_{12}C_{23}C_{16} + C_{11}C_{23}C_{26} + C_{36}C_{12}^2 + C_{22}C_{13}C_{16}$,

$A_{18} = C_{11}C_{22}C_{35} + C_{12}C_{13}C_{25} + C_{12}C_{23}C_{15} - C_{11}C_{23}C_{25} - C_{35}C_{12}^2 - C_{22}C_{13}C_{15}$,

$A_{19} = -C_{11}C_{22}C_{34} - C_{12}C_{13}C_{24} - C_{12}C_{23}C_{14} + C_{11}C_{23}C_{24} + C_{34}C_{12}^2 + C_{22}C_{13}C_{14}$,

$A_{20} = C_{11}C_{22}C_{33} + C_{12}C_{13}C_{23} + C_{12}C_{13}C_{23} - C_{11}C_{23}^2 - C_{33}C_{12}^2 - C_{22}C_{13}^2$.

The elastic constants of int model can be obtained by theoretical calculation as follows:

$$C_{\text{int}} = \begin{pmatrix} 86.138 & 53.252 & 54.007 & 1.614 & 0.267 & -2.713 \\ 53.252 & 88.327 & 54.647 & 2.124 & 2.529 & 0.184 \\ 54.007 & 54.647 & 83.672 & -3.625 & -3.672 & -1.728 \\ 1.614 & 2.124 & -3.625 & 32.738 & -1.386 & 5.226 \\ 0.267 & 2.529 & -3.672 & -1.386 & 37.284 & 1.442 \\ -2.713 & 0.184 & -1.728 & 5.226 & 1.442 & 32.056 \end{pmatrix} \qquad (15)$$

The elastic constants of int model satisfy the criteria in formula (14), so int model is also mechanically stable.

Supplementary References

1. Dong, W.-D. et al. Phase conversion accelerating “Zn-Escape” effect in ZnSeCFs heterostructure for high performance sodium-Ion half/full batteries. *Small* **18**, 2105169 (2022).
2. Li, J. et al. Rationally designed ZnTe@C nanowires with superior zinc storage performance for aqueous Zn batteries. *Small* **19**, 2304916 (2023).
3. Huang, M. et al. TiO_2-based heterostructures with different mechanism: a general synergistic effect toward high-performance sodium storage. *Small* **16**, 2004054 (2020).
4. Zheng, J., Yan, P., Estevez, L., Wang, C. & Zhang, J. G. Effect of calcination temperature on the electrochemical properties of nickel-rich $LiNi_{0.76}Mn_{0.14}Co_{0.10}O_2$ cathodes for lithium-ion batteries. *Nano Energy* **49**, 538-548 (2018).
5. Sedaghati-Jamalabad, G. Bagheri-Mohagheghi, M. High specific surface area (2840.3 m^2/g) in activated carbon/$SnFe_2O_4$ spinel nanocomposite: structural, optical, dielectric, and magnetic properties for Li-ion battery soft and porous electrode applications. *Applied Physics A* **130**, 645 (2024).
6. Mouhat, F. & Coudert, F.-X. Necessary and sufficient elastic stability conditions in various crystal systems. *Phys. Rev. B* **90**, 224104 (2014).
7. Gao, J. et al. Criteria of mechanical stability of seven crystal systems and its application: taking silica as an example. *Chin. J. High Pressure Phys.* **36**, 051101 (2022).
8. Sun, D., Liu, K., Hu, J. & Zhou, J. Antiblocking heterostructure to accelerate kinetic process for Na–ion storage. *Small* **17**, 2006374 (2021).

9. Jiang, Y. et al. Confining $CoTe_2$–ZnTe heterostructures on petal–like nitrogen–doped carbon for fast and robust sodium storage. *Chem. Eng. J*. **451**, 138430 (2023).

10. Dong, C. et al. Willow–leaf–like ZnSe@N–Doped carbon nanoarchitecture as a stable and high–performance anode material for sodium–ion and potassium–ion batteries. *Small*, **16**, 2004580 (2020).

11. He, Y. et al. In–situ rooting ZnSe/N–doped hollow carbon architectures as high–rate and long–life anode materials for half/full sodium–ion and potassium–ion batteries. *Energy Storage Mater.* **23**, 35-45 (2019).

12. Huang, D. et al. One–dimensional ZnSe@N–doped carbon nanofibers with simple electrospinning route for superior Na/K–ion storage. *Chin. Chem. Lett.* **34**, 107416 (2023).

13. Men, S. et al. Unraveling the stabilization mechanism of solid electrolyte interface on ZnSe by rGO in sodium ion battery. *J. Energy Chem*. **54**, 124-130 (2021).

14. Yu, L. et al. Trash to treasure: carbon–free ZnSe derived from waste zinc foil as a high–rate and long–life anode material enabling fast–charging sodium–ion batteries. *J. Power Sources* **542**, 231801 (2022).
